# Epidemiology of influenza over a ten-year period in Belgium: overview of the historical and current evidence

**DOI:** 10.1186/s12985-023-02238-1

**Published:** 2023-11-21

**Authors:** A. Prezzi, X. Saelens, D. Vandijck

**Affiliations:** 1 Department of Public Health and Primary Care, Faculty of Medicine and Health Sciences, Ghent University, Corneel Heymanslaan 10, 9000 Ghent, Belgium; 2 Department of Biochemistry and Microbiology, Faculty of Sciences, Ghent University, K. L. Ledeganckstraat 35, 9000 Ghent, Belgium; 3 Flanders Institute for Biotechnology - UGent Center for Medical Biotechnology, Technologiepark 927, B-9052 Ghent (Zwijnaarde), Belgium; 4 Belgian Poison Control Center, Bruynstraat 1, 1120 Brussels, Belgium

**Keywords:** Influenza, Flu, Epidemiology, Vaccination, Hospitalisation

## Abstract

**Background:**

Generally influenza, a contagious respiratory disease, leads to mild illness, but can present as a severe illness with significant complications for some. It entails significant health challenges and an economic burden. Annual vaccination is considered the most effective preventive measure against influenza, especially in high-risk groups.

**Method:**

Epidemiological, demographic and vaccination data of influenza from 2009-to-2019 is collected from Sciensano, the Belgian Institute for Health. Sciensano monitors influenza virus through two surveillances: the Influenza-Like Illness (ILI) surveillance in primary care and the Severe Acute Respiratory Infections (SARI) surveillance in hospital settings.

**Results:**

49.6% [± 8.5] of all ILI-samples tested positive in this period. Influenza A was the dominant circulating type, accounting for 73.7% [± 27.5] of positive samples, while influenza B accounted for 24.3% [± 26.7]. For SARI-surveillance, the average rate of samples tested positive was 36.3% [± 9.3]. Influenza A was responsible for respectively 77.7% [± 23.8] of positive samples and influenza B for 22.2% [± 23.7]. Since 2010, epidemics typically lasted about 9.3 weeks [± 2.7]. From 2012 to 2019 the average vaccine effectiveness was 34.9% [± 15.3].

**Conclusion:**

Influenza is quickly considered a trivial disease, but can have substantial repercussions. It remains difficult to identify the level of treat of influenza due to antigenic evolution. Measures to prevent, control and treat are needed. Vaccines that provide broader and more durable protection that can be produced more rapidly could be a potential solution.

## Introduction

Influenza entails significant health challenges and annual costs for our healthcare (system) [1, 2]. It is a contagious respiratory disease that is generally mild, but can present as a severe illness with complications for some [1, 3]. In order to address the challenges at hand, there remains a significant opportunity for the development and implementation of innovative preventive and curative measures, which can be targeted towards both the general population and specific groups [4, 5].

### Influenza viruses

Influenza A, B and C viruses cause human influenza [6, 7]. Influenza A can be subdivided into subtypes based on the genetic and antigenic properties of hemagglutinin (HA) and neuraminidase antigens (NA). The most significant challenge of influenza A is its ability to jump species barriers and undergo fast evolution, which can result in new subtypes and potential pandemics [6, 8]. Influenza B is subdivided into two antigenic lineages: Yamagata and Victoria [9]. Both lineages are known to co-circulate globally. Influenza B viruses generally exhibit slower rates of genetic and antigenic evolution than influenza A viruses [6]. Both influenza A and B can mutate and spread effectively, causing annual seasonal epidemics in moderate climate zones and prevail year round in tropical regions [7, 9]. Influenza C generally leads to mild illness and is not typically associated with epidemics. Neither influenza B nor C have been implicated in previous pandemics [6, 8].

HA and NA are crucial for the pathogenicity and transmission of influenza A and B viruses. HA plays a critical role in the virus’ ability to infect its host by enabling attachment to specific receptor sites on the respiratory cells surface. It is responsible for initial interaction between the virus and the host cell and its successful binding is a prerequisite for viral replication. NA facilitates the fusion of influenza virus with the host’s respiratory cells, which enables the release of newly formed virus particles from infected cells. Once virus particles have been released, they can enter and replicate within other cells, leading to the infection spreading [8]. There are 18 HA and 11 NA subtypes which occur in nature in different combinations [6, 7].

### Antigenic evolution

Antigenic evolution is the result of genetic mutations in HA and NA. These antigens are the targets of the host’s immune system and are the main components of influenza vaccines. Antigenic evolution occurs through two types of mechanisms: antigenic drift and antigenic shift [10].

Antigenic drift is characterised by smaller gene changes that lead to mutations in antibody-binding sites of surface proteins HA and NA [10, 11]. These changes occur repeatedly as the virus replicates, resulting in influenza viruses that have similar antigenic characteristics that can still be recognized by antibodies created by the immune system. However, these changes eventually accumulate, resulting in antigenically different viruses over time [10]. New variants replace older ones, allowing the virus to spread more easily among hosts as the immune system’s antibodies cannot effectively inhibit them. This can lead to more severe influenza epidemics and is the primary reason why people can get re-infected with the influenza virus [11].

Antigenic shift, which is a big sudden change in influenza A, can lead to new subtypes with novel HA and/or NA proteins originating from mammalian or avian sources or that have not been circulating under humans in decades [10, 11]. This results in increased transmission and more severe illness, complications and deaths due to lack of immunity [10–12]. As antigenic drift happens constantly, antigenic shift happens less frequent (about three times every 100 years) [10, 11]. After undergoing an antigenic shift, influenza viruses still evolve by antigenic drift. [11].

### Annual vaccination

The most effective preventive measure against influenza is annual vaccination, especially at high-risk groups [1, 4, 13]. Vaccine composition is based on available data regarding the most common virus strains, antigenic drifts and predictions [11, 14, 15]. To effectively control viruses, vaccines require closely matched or similar antigenic profiles to circulating strains [11]. This emphasizes the importance of annual revaccination for high-risk groups, as strain-based vaccines offer limited protection beyond one year [4]. Administering vaccinations between mid-October and mid-December maximises effectiveness, as the vaccine takes about two week to take effect [16].

Influenza vaccines may have limited effectiveness due to a mismatch between circulating viruses strains and vaccine composition [11]. 90% of influenza vaccines are produced using embryonated eggs, which can take up five to six months before vaccines can be delivered [4, 11]. A significant antigenic drift can prevent production of a vaccine corresponding to the new circulating strain, reducing vaccine efficacy and potentially leading to an epidemic outbreak [11].

### Antiviral resistance

Seasonal vaccination is the primary preventive measure against influenza, while antiviral treatment can supplement management efforts, particularly for hospitalised patients or during pandemics when a suitable vaccine is unavailable [17–19].

Currently, three main groups of antiviral drugs are available. Neuraminidase inhibitors, like oseltamivir and zanamivir, are effective against influenza A and B viruses. M2 ion channel inhibitors, such as amantadine and rimantadine, prevent influenza A replication [17, 20]. Baloxavir marboxil, or Xofluza, inhibits the replication of four influenza virus types [21]. Phenotypic resistance to these antivirals can emerge, often via target protein mutations, rendering the drugs less or non-effective. The World Health Organization (WHO) has reported an increase in resistance cases since 2007 [20]. Xofluza maintains efficacy against neurominidase inhibitor-resistant viruses [21]. Currently, most circulating influenza viruses are resistant to M2 ion channel blockers [17, 20].

### Economic impact

The magnitude of the economic challenges posed by influenza is contingent upon the severity of the epidemic and the scale of the influenza-infected population [1, 14]. Its economic burden encompasses both direct and indirect costs. Direct costs are an inherent consequence of influenza, including expenses associated with (para)medical visits and hospitalisations in case of severe illness [2, 13, 14]. Indirect costs, such as lost productivity due to presenteeism and absenteeism, also have a considerable impact [4].

Influenza virus poses a substantial economic burden on society. In 2017, the majority of healthcare expenses, amounting to 77%, were covered by government funds, which primarily consisted of compulsory health insurance schemes [22, 23]. These funds are sourced through the imposition of taxes on the general population [23]. Additionally, citizens themselves contributed directly to healthcare expenses by making co-payments, which accounted for 18% of the total healthcare expenditure. The remaining 5% was covered by voluntary health insurance programs [22]. The economic impact of influenza is also significant in the United States, where it represents 65% of the total estimated economic burden attributed to all vaccine-preventable diseases [24].

The WHO has set the guideline of a 75% vaccination rate for the elderly [25, 26]. Yet in 2018, 58% of individuals aged 65 years or older were vaccinated in Belgium [22]. This is a decline from the 63.6% vaccination rate reported in 2009 [26]. Although the set target by WHO has not been met, Belgium still surpasses the average vaccination rates observed across Europe [22, 26]. This observed decline in vaccination rates is a critical threat to economic, and medical, outcomes associated with influenza. Vaccinations are among the most cost-effective public health measures and the best prevention strategy. It is imperative that sustainable and effective vaccination programs be implemented and strengthened throughout Europe in order to address the issue [27].

## Method

This study has collected current epidemiological and demographic data on the influenza virus and its vaccine from the Sciensano database. As the Belgian Institute for Health, Sciensano monitors the health of the population and utility animals on a daily basis. As such, its responsible for collecting comprehensive information on influenza virus activity and the individuals affected by it in Belgium. Sciensano plays a critical role in informing national policies and programs to promote public health and improve the well-being of the Belgian population [28]. Two surveillances are conducted to ensure effective monitoring of the influenza virus and associated vaccination. Influenza-Like Illness (ILI) surveillance is a network of sentinel general practitioners (GPs) who provide clinical and virologic follow-up of flu activity. Since 2007, ILI data has been collected in primary care, which includes all individuals presenting with flu-like symptoms based on specific criteria, including sudden onset of symptoms, high fever and respiratory and systemic symptoms. GPs use a standardised form to report clinical outcomes, age range, hospitalisation, antiviral therapy, vaccination status and number of acute respiratory infections (ARI). Additionally, they obtain two nasopharyngeal swabs per week to yield virological information. Since 2010, Severe Acute Respiratory Infections (SARI) surveillance has been established to obtain more comprehensive insights regarding severity and virulence of circulating influenza viruses. It is comprised of a network of six hospitals that gather data during the epidemic phase of seasonal influenza. Healthcare workers collect clinical data and nasopharyngeal swabs from every patient with SARI. The criteria utilised to determine a SARI encompass a manifestation onset within seven days, fever of ≥ 38 °C, cough or dyspnea that necessitated hospitalisation for at least 24 h [29–31].

Both surveillance systems are complementary to each other. Primary aim is to detect an influenza epidemic or pandemic, evaluate their intensity, severity and duration, determine circulating viruses and aid in decision-making processes for next-season influenza vaccine strains [29, 31]. Each year, representative strains were chosen for sequencing to gain insights into viral evolution and to establish appropriate reference materials for candidate vaccine strains. Results are collected in multiple reports per year. Of these reports seasonal influenza surveillance, weekly influenza bulletins, virological surveillance of influenza in Belgium and epidemiological surveillance of influenza infections are included in this study. These are selected based on specific inclusion criteria: influenza virus, influenza vaccination status, influenza hospitalisations, epidemiological and demographic influenza data, influenza data from Belgium and influenza data from 2009 to 2019. The reports discuss various aspects of influenza, such as length and intensity of epidemic/pandemic, when epidemic threshold is crossed, incidence of GP visits, results of ILI- and SARI-surveillances, dominant (sub)type/lineage, characterisation of viruses, vaccine composition and effectiveness, antiviral monitoring and hospitalisations. Sciensano obtained vaccine effectiveness estimates through a test-negative design case–control study, accounting for age, gender, sampling month, underlying medical conditions and surveillance program, using data from both surveillance sources [31].

Collected information from the surveillance reports is summarised and visualised in tables and figures. Appropriate statistics, such as percentages, means and standard deviations (SD), are utilised to provide an overview of the data. In Table 1, the mean and SD are calculated with and without the 2009 pandemic as this season’s data is considered exceptional. The statistical analysis is performed using SPSS Statistics 29 software.

**Table 1:** Epidemiological data of the influenza virus from 2009 to 2019 in Belgium

	2009 pandemic	2010 2011	2011 2012	2012 2013	2013 2014	2014 2015	2015 2016	2016 2017	2017 2018	2018 2019	Mean	SD
Length (weeks)	33	12	6	12	6	10	10	7	12	8	9.3 (11.7)	2.7 (7.9)
Period (week/year)	40/2009; 20/2010	50/2010; 9/2011	6/2012; 11/2012	1/2013; 12/2013	6/2014; 11/2014	4/2015; 13/2015	4/2016; 13/2016	2/2017; 9/2017	2/2018; 13/2018	4/2019; 11/2019	8.8/11.3^**a**^	15.6/1.6^**a**^
											11.9/12.2*^a^	12.2/3.1*^a^
Intensity (Low-Moderate-High)	M	M	M	H	L	H	M	M	M	M	M	**/**
Intensity (maximum #GP consultations/100.000 citizens)	–800	500	531	1100	311	1036	775	787	744	761	727.2	251.2
											734.5*	238*
Intensity (week/year of maximum #GP consultations)	44/2009	1/2011	7/2012	6/2013	9/2014	6/2015	9/2016	5/2017	10/2018	7/2019	6.7	2.7
											10.4*	12.1*
ILI-Surveillance: positive samples (%)	37.2	48.8	42.6	59	34.5	51.9	51.6	51.5	59	52.7	51.1	7.6
											49.6*	8.5*
Positive influenza A samples (%)	99.5	55.4	93.4	44.4	98.2	83.7	48.8	99.7	34.6	99.2	70.5	27.5
											73.7*	27.5*
A(H3N2) (%)		2	96	20.2	58.6	80.2	1.1	97.3	25.4	72.5	50.4	38.8
A(H1N1)pdm09 (%)	54	98	0.09	75	38.6	14.8	93	0.9	69.4	23.6	45.9	38.7
											46.7*	36.6*
Positive influenza B samples (%)	0.05	44.6	6.6	55.3	1.6	16.3	51.7	0.3	65.4	0.8	27	26.9
											24.3*	26.7*
B/Yamagata (%)		7	80	95.4	50	92.4	3	100	92.9	0	57.8	43.4
B/Victoria (%)		93	12	4.1	25	7.6	96.4	0	3.2	100	37.9	44.5
First positive samples (week/year)	28/2009	**/**	46/2011	45/2012	46/2013	40/2014	42/2015	47/2016	47/2017	48/2018	45.2^a^	2.6^a^
										43.5*^a^	6*^a^	
Increased positive samples (week/year)	34/2009	48/2010	3/2012	52/2012	4/2014	50/2014	53/2015	53/2016	52/2017	52/2018	40.8^a^	21.2^a^
											40.1*^a^	20.1*^a^
Positivity rate (week:%)	**/**	3:73	7:73	5:85	10:60	5:74	10:80	5:74	7:88.7	10:82	6.9:76.6	2.6:8.5
Sari-surveillance: positive samples (%)	/	18.7	**31.3**	**43.1**	**30.7**	**46.2**	**46**	**39.6**	**41.5**	**29.7**	**36.3**	**9.3**
Positive influenza A samples (%)	**/**	61.5	98	52.7	99.3	88.3	62.4	98.8	39.1	99.7	77.7	23.8
A(H3N2) (%)	/	2	94	29.5	60.2	82.7	1	94.6	27.6	79.9	52.4	38.1
A(H1N1)pdm09 (%)	/	98	0	66	31	11.5	90.5	0.4	67.8	18.1	42.6	38.4
Positive influenza B samples (%)	**/**	38.5	2	47.1	0.6	11.7	36.8	1.2	60.9	0.3	22.2	23.7
B/Yamagata (%)	/	12.9	100	93	100	92.4	1.1	71.4	96.5	50	68.4	38.5
B/Victoria (%)	/	87.1	0	2.5	0	6.4	97.8	28.6	1.3	50	29.7	39.6
Hospitalisation: LOS (days)	**/**	**/**	9.1	8.7	7.7	9.4	6.8		8.6	8.8	8.4	0.9
Complications (%)	/	/	16	13^b^–15^c^	15	14.1	11.6	14^b^	11.3^b^	13^b^	13.6	1.7
Deaths (%)	/	/	8.6	4	5.7	6.7	5	6	5.6	6.4	6	1.3
Patients receiving antiviral treatment (%)	6.3^d^	/	8	15	5	3	3	/	/	/	6.8	5
Seasonal Influenza virus	A(H1N1)pdm09	A(H1N1)pdm09 & B/Vic	A(H3N2)	A(H1N1) **pdm09 & B/Yam**	A(H3N2)& A(H1N1) **pdm09**	A(H3N2) & B/Yam	A(H1N1) **pdm09 & B/Vic**	A(H3N2)	B/Yam & A(H1N1) **pdm09**	A(H3N2)& A(H1N1) pdm09		

The first given mean and SD are calculated without the 2009 pandemic

*Mean and SD are calculated with the 2009 pandemic The Victoria lineage is abbreviated to B/Vic and the Yamagata lineage is abbreviated to B/Yam

^a^Only weeks measured

^b^Only influenza positive individuals included

^c^All SARI-cases included

^d^Antiviral treatments by only GPs

## Results

This paper presents a chronological description of demographic data coupled with epidemiological data and complemented by information on vaccinations across seasons.

### 2009 Pandemic

In April 2009, a new variant of influenza A, identified as A(H1N)pdm09, was first reported in Mexico and subsequently in the United States of America. Belgium implemented a high-risk case identification program targeting travellers from countries with high incidence of A(H1N)pdm09 as a containment strategy. The primary aim was to limit the spread by testing and isolating high-risk individuals. The WHO confirmed this outbreak as a pandemic in June. In response, Belgium implemented a control strategy in July by strengthening the network of GPs and noting and reporting every patient who met the influenza case definition and sought medical attention from GPs, local care hotlines or hospitals on a daily basis. This strategy aimed to reduce the number of patients, complications and fatalities through rapid case management [20, 32, 33].

The 2009 flu season, caused by A(H1N1)pdm09 resulting from an antigenic shift, began when the epidemic threshold was crossed in week 40/2009. GP consultation rates exceeded the threshold again in week 49/2009, indicating the end of the epidemic, although the season only concluded in week 20/2010. Children <15 years old were mainly affected, with those <five years having the highest incidence of ARIs. Seniors were relatively spared. GP consultations for ARIs were higher than during an average epidemic. 6.3% of patients received antiviral treatment, with mainly children <five years (8.3%) and seniors (12.2%) treated. The end of this season saw only limited diagnoses of influenza B lineages Victoria and Yamagata [20, 32].

This seasons vaccine composition was determined prior to the pandemics announcement. Majority of circulating influenza B viruses belonged to B/Victoria which included vaccine strain B/Brisbane/60/2008 was based on. The vaccine also contained inactive antigens for A(H3N2), but did not provide protection against new subtype A(H1N1)pdm09. This necessitated the rapid development of a new vaccine by the WHO, which identified A/California/07/2009 as an (anti)genetically similar strain to A(H1N1)pdm09. Besides this strain, it also contains the immunostimulating adjuvant AS03 that helps antigens to elicit early, high and long-lasting immune response with less antigen, thus saving on vaccine production cost [34]. This resulted in two vaccines: a seasonal trivalent vaccine and Pandemrix A(H1N1)pdm09 vaccine (see Table 2). The vaccination campaign for the seasonal vaccine commenced on the first of October 2009. It aimed at reducing complications such as hospitalisations and deaths, particularly among elderly and other high-risk groups. The campaign for Pandemrix vaccine started on October 19^th^ 2009 and continued until March 31^st^ 2010. A total of 733,025 doses were administered mainly at end of this epidemic, owing to its late availability and an early pandemic. The objectives were to maintain healthcare activities, limit A(H1N1)pdm09-related complications and ensure continuity of educational system. So hospital employees, extramural medical and teaching staff and high-risk individuals were the primary target groups, with no age criteria applied. From W42/2009 to W50/2009 GPs registered seasonal vaccination status of their ILI-patients. A greater portion of ILI-patients were vaccinated than in the general population. From W46/2009 to W50/2009, GPs registered Pandemrix vaccination status of their ILI-patients. Fewer vaccinated adults with ILI were observed compared to the general population. There was one H275Y-mutation detected in an immunosuppressed patient infected with A(H1N1)pdm09 and treated with oseltamivir [20].

**Table 2:** Data about the influenza vaccination from 2009 to 2019 in Belgium

	’09–’10 seasonal vaccine	’09–’10 Pandemrix vaccine	’10– ‘11	’11– ‘12	’12–‘13	’13–‘14	’14–‘15	’15–‘16	’16–‘17	’17–‘18	’18–‘19	Mean [SD]
Composition	A/Brisbane/59/2007	A/California/7/2009	A/California/7/2009	“	“	“	“	“	“	A/Michigan/45/2015	“	
A(H1N1)pdm09		AS03										
A(H3N2)	A/Brisbane/10/2007	/	A/Perth/16/2009	“	A/Victoria/361/2011	A/Texas/50/2012	“	A/Switzerland/971529/2013	A/Hong-Kong/4801/2014	“	A/Singapore/INFIMH-16–0019/2016	
B	B/Brisbane/60/2008^a^	/	“	“	B/Wisconsin/1/2010^b^	B/Massachusetts/2/2012^b^	“	B/Phuket/3073/2013^b^	B/Brisbane/60/2008^a^	“	B/Colorado/06/2017^a^	
Quadrivalent	/	/	/	/	/	/	/	B/Brisbane/60/2008^a^	B/Phuket/3073/2013^b^	“	“	
Effectiveness (%)	/	/	Moderate to high	Low to moderate	47	55	19	41	28^**c**^	41	13	34.9 [15.3]
Antiviral resistance	Yes^**d**^	/	No	/	No	Yes^**d**^	No	Yes^**d**^	No	No	Yes	

^a^B/Victoria strain

^b^B/Yamagata strain

^c^Mutation in influenza strain identified

“Same vaccine strain as year before

### 2010–2019 epidemics

**Fig. 1:**
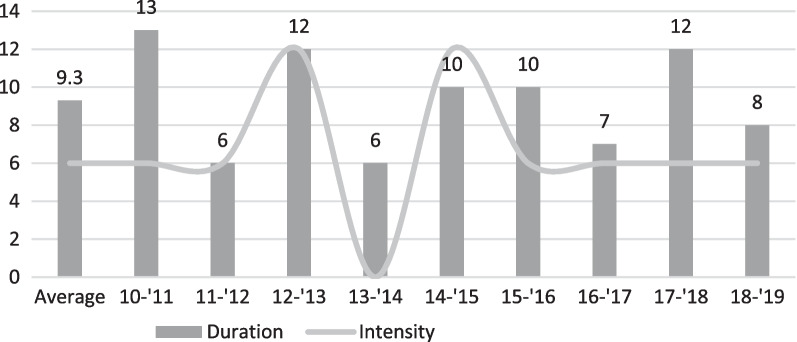
Duration and intensity epidemics 2010–2019. X-axis represents the average of seasons 2010–2019 and these seasons separately. Y-axis represents the duration in weeks and the intensity as: low intensity = 0, moderate intensity = 6, high intensity = 12

From season 2010 to 2019, the co-circulation of A(H1N1)pdm09, A(H3N2), B/Yamagata and B/Victoria was observed for varying lengths, ranging from six to 13 weeks. Moderate intensity was noted in 66.7% of the seasons, high intensity in 22.2% and low intensity in 11.1% (Fig. 1). There were more positive samples detected by ILI-surveillance than observed by SARI-surveillance. Influenza A was predominantly observed in both surveillances. When analysed separately, A(H3N2) and B/Yamagata were observed to be more circulating. To determine the level of match between circulating viruses and vaccine strains, sequencing was conducted on all seasonal vaccines. From 2012 to 2019, the average vaccine effectiveness was 34.9% [SD:15.3] (Table 2).

Season 2010–2011 was characterized by cocirculation of influenza A and B. In primary care, infants and children aged up to 14 years were most frequently diagnosed with influenza, while hospitals mainly detected influenza in individuals aged 15–65 years [35, 36]. All 32 sequenced A(H1N1)pdm09 strains were (anti)genetically similar to vaccine strain A/California/7/2009.

For A(H3N2), two strains were sequenced: one matched vaccine strain A/Perth/16/2009 and the other matched A/Hong Kong/2121/2010. All 27 sequenced B/Victoria strains were similar to vaccine strain B/Brisbane/60/2008, and four sequenced B/Yamagata strains were similar to strain B/Bangladesh/3333/07. The majority of circulating viruses were well matched with the seasonal vaccine [35].

In 2011–2012 the epidemic began later and was shorter than usual. The virus predominated in children <five years who consulted their GP the most. Children aged five to 14 also had a high number of GP visits; while those aged 15–64 and elderly visited their GP the least. Hospitals collected more respiratory samples from adults (60%) than from children (40%). Adults were more affected by influenza viruses than other respiratory viruses, in contrast to children. In both surveillances some samples could not be (sub)typed due to low viral load. A(H3N2) was the predominant virus, with 86% of all analysed samples containing this subtype [37, 38]. Multiple A(H3N2) variants were circulating, different from vaccine strain A/Perth/16/2009. B/Yamagata was the most common strain, while the vaccine included a B/Victoria-lineage. So the circulating viruses were less covered by the vaccine [37].

During the season 2012–2013, co-infections with multiple subtypes/lineages of influenza A and B were detected in the ILI-surveillance. Children from five to 14 years were the most affected by influenza (74%), while elderly ≥ 65 years were the least affected (42%). In the 5–14 age group, influenza B was dominant, whereas influenza A and B co-circulated in other age groups. A(H1N1)pdm09 affected mostly individuals ≥ 15 years (69%) in comparison to children <15 years old (52%) in primary care. The hospitalisation rate for patients with ILI was 0.4%. The positivity rate of SARI-samples are lower in children <five years old. Both influenza A and B were detected in this group with influenza A being more common. The positivity rate was higher in children aged 5–14, who were mainly infected with influenza B. A(H1N1)pdm09 affected mostly children <15 years (38%) in comparison to individuals ≥ 15 years (32%) in hospitals. B/Yamagata was more prevalent in children <15 years (51%) compared to individuals ≥ 15 years (41%). Majority of hospitalised patients were adults with a median age of 68 years (63%), while 37% were children <15 years with a median age of two years. Genders distribution was 47% women and 53% men. 92% had a fever before or upon admission, and the most common symptoms were fever and coughing. High-risk patients were less likely to cough, but more likely to have dyspnoea. Symptoms also varied with age: dyspnoea was more common in elderly, while other symptoms were more common in children <15 years. A critical condition was reported significantly more in elderly. Among all hospitalised patients, 68% had one or more risk factors. Antiviral treatment was significantly more used for patients ˃15 years, influenza positive and with risk factors. The positivity rate of samples infected with influenza was lower in the SARI-surveillance (43%) than in the ILI-surveillance (67%). In both surveillances, some samples could not be (sub)typed due to low viral load [39–41]. There was significant difference in vaccination proportion in SARI-patients: 35% of influenza negative patients was vaccinated, while this was 28% in positive influenza patients. An adjustment to the vaccine composition was made for A(H3N2) and B/Yamagata-lineage was added instead of B/Victoria. The utilised vaccine strain reacted well to all sequenced A(H1N1)pdm09 samples. Ten of 11 sequenced A(H3N2) samples had a lower reactivity to the egg-propagated vaccine strain, despite being close to A/Victoria/361/2011. A(H3N2) isolates belonged to cell-propagated reference strain A/Victoria/208/2009. Two of 22 B/Yamagata samples represented B/Wisconsin/1/2010 and these showed reduced reactivity to this vaccine strain. So there was only a good match between circulating viruses and the vaccine for A(H1N1)pdm09. The vaccine worked better in children [5–15] and young adults [15–45].

The epidemic of 2013–2014 started later and had a shorter duration compared to previous seasons. GPs mainly collected samples from individuals aged 15 to 44 years. The positivity rate was similar among all age groups; ranging from 30 to 50%. Influenza A subtypes did not vary across age groups. In primary care, A(H3N2) was predominant in children and adults. SARI-surveillance collected most samples from children <five years, and their positivity rate was significantly lower (21%). Across other age groups the positivity rate was comparable (30–40%). Influenza A subtypes distribution was similar across age groups, with A(H3N2) accounting for 52% in children ˃15 and 64% in adults. Among all severe influenza cases, 47.5% were attributed to influenza A, with 14.3% A(H1N1)pdm09 and 82.1% A(H3N2). No samples were positive for influenza B. In both surveillances, non-subtypeable samples were due to low viral load [42, 43]. Vaccine strain A/California/7/2009 was used for subtype A(H1N1)pdm09, which was a good match with all examined reference strains. Changes were made for A(H3N2) and B/Yamagata. Reference strains for A(H3N2) were antigenically similar to each other and vaccine strain A/Texas/50/2012. Despite poor reactivity against egg-propagated vaccine strain A/Texas/50/2012, A(H3N2) reacted well against genetically similar cell-propagated A/Victoria/361/2011. No significant antigenic drift was detected between circulating A(H3N2) viruses and original A(H3N2) vaccine strain. Some antigenic difference between cell-grown and egg-propagated viruses have been detected due to mutations induced in H3N2-strain during vaccine production on eggs. This season, influenza B had low circulation and B/Yamagata belonged to B/Wisconsin/1/2010. The proportion of vaccinations in SARI-patients differenced slightly: 29% of influenza negative patients and 28% of positive influenza patients were vaccinated [43]. In 2013, 56.4% of elderly ≥ 65 years were vaccinated in Belgium, which is close to the European Union average (55%), but below the target of 75%. Vaccination status differed per region for elderly: 60.6% in Flanders, 50.1% in Wallonia and 47.8% in Brussels. Residential facilities were often associated with a higher rate of influenza vaccinations and more contact with GPs [44].

During the beginning of season 2014–2015, influenza A dominated with a predominance of A(H3N2). Since week 10/2015, influenza B has taken over. Samples collected by GPs were mainly from individuals aged 15–44 years. The positivity rate varied significantly across age categories, with fewer positive samples detected in 15–44 year age group in comparison to those aged 5–14 and 45–65 years. A(H3N2) was found predominant in children and elderly, but there were relatively more A(H1N1)pdm09 cases detected in adults aged 15–64 years. No age differences were identified for influenza B. ILI-surveillance detected two samples (0.5%) that were co-infected with A(H3N2) and A(H1N1)pdm09. SARI-surveillance positivity rate peaked in week 6/2015 with 43%. The highest peak (70%) was observed in patients ˃85 years old. Samples were mainly collected in children <four years old and elderly aged 65–84 years. Children were less frequently positive than elderly persons. There were no co-infections of influenza A and B in adults. A(H3N2) was predominant in hospitals. There were significantly fewer A(H1N1)pdm09 positive patients in age group ˃85 years than in younger influenza patients. In both surveillances some samples could not be subtyped due to low viral load. Samples from GPs (62%) were significantly more positive than those from hospitals (46%), and the predominant virus, A(H3N2), was more common in SARI-surveillance (77%) than in ILI-surveillance (70%). SARI-patients were less positive for both influenza B (10,8% vs 11,4%) and A(H1N1)pdm09 (12% vs 18%), but the differences in subtypes were not statistically significant [45, 46]. There were no changes made in the vaccine composition. All eight sequenced A(H1N1)pdm09 samples were antigenically similar to vaccine strain A/California/7/2009. Approximately 60% of A(H3N2) viruses circulating in Belgium were antigenically different from vaccine strain A/Texas/50/2012. All sequenced B/Yamagata samples belonged to reference strain B/Phuket/3073/2013, whereas the chosen vaccine strain B/Massachusetts/02/2012 did not recognize most circulating B viruses well [45].

In season 2015–2016 GPs estimated that 380,000 Belgians experienced symptomatic flu infection, with one-third being children, which was a higher fraction than in the previous five years. Most samples were taken from age 15 to 44 years old and least from elderly ˃85 years, of those two were co-infected with both influenza A and B. High-risk adults aged 45 to 64 years were more severely affected. Adults from 15 to 64 years had fewer positive samples than children <15 years and elderly aged 65 to 84 years. Influenza (sub)types varied with age: B/Victoria being predominant in children <15 years, while A(H1N1)pdm09 was predominant in adults ˃45 years. There were no major differences among age group 15–45 years old. In SARI-surveillance a peak positivity rate of 30% was observed in week 8/2016., with most samples collected from children <five years and elderly aged 65 to 84 years. Adults aged 45 to 64 years were significantly less likely to test positive. B/Victoria was predominant in children between 5 and 14 years, while A(H1N1)pdm09 co-circulated with B/Victoria with a slight predominance of A(H1N1)pdm09 in the other age groups. Out of all severe flu cases, 65.4% were infected with influenza, with 49.1% infected with influenza A (88.9% A(H1N1)pdm09, 7.4% A(H3N2) and 3.7% non-subtypeable). In general, GP samples (61%) were significantly more positive than hospital samples (46%). A(H1N1)pdm09 was significantly more common in SARI-patients (62%) than in ILI-patients (48%) during SARI-surveillance period. In both surveillances some samples could not be (sub)typed due to low viral load [47, 48]. For the first time in Belgium, a quadrivalent vaccine was available including both B/Yamagata and B/Victoria. All A(H1N1)pdm09 samples were antigenically similar to vaccine strain. 75% of sequenced A(H3N2) samples were closer to reference strain A/Hong Kong/5738/2014 and only 25% were closer to used vaccine strain /Switzerland/971529/2013. Only a limited number of A(H3N2) and B/Yamagata viruses circulated this season. All B/Yamagata samples were similar to B/Phuket/3073/2013 and all B/Victoria samples to B/Brisbane/60/2008. This seasons trivalent vaccine included B/Yamagata, which was a mismatch with B/Victoria being dominant. The vaccine effectiveness was on average 41%, 50% for A(H1N1)pdm09 and 32% for influenza B. The distribution of the administered vaccine type is unclear. There was one H275Y-mutation detected in an immunodeficient patient treated with oseltamivir [47].

In 2016–2017, there were an estimated 490,000 ILI-consultations and 280,000 lab-confirmed clinical influenza virus infections, yielding a positivity rate of 74%. Majority of samples were obtained from individuals aged 15–64 years, while limited number of samples were collected from children <five years and elderly ˃85 years. Children between five and 14 years old were the most affected age group. Elderly aged ≥ 65 years were more affected compared to previous seasons. During SARI-surveillance, a peak positivity rate of 55.5% was observed in week 6/2017, with 60% of the samples collected and tested positive belonging to elderly aged 65–84 years. Among severe influenza cases, 71.2% were infected with influenza A, and individuals aged ≥ 65 with respiratory comorbidities were found to be at higher risk of developing complications and death. In both surveillances, (sub)types did not vary with age and low viral load hindered the (sub)typing of some samples. In general, samples from GPs (60.2%) were significantly more likely to test positive than samples from hospitals (39.5%) during SARI-surveillance period [30, 49]. Also during this season, a quadrivalent vaccine was available that included B/Yamagata. All sequenced A(H1N1)pdm09 samples were antigenically similar to vaccine strain A/California/7/2009. A(H3N2) was the predominant subtype, and in 61.1% of the cases belonged to a clade antigenically similar to vaccine strain A/Hong Kong/4801/2014, with the remaining 38.9% belonging to a clade represented by A/Hong Kong/4801/2014. The circulating B/Victoria virus was represented by vaccine strain B/Brisbane/60/2008. All B/Yamagata samples were represented by quadrivalent vaccine strain B/Phuket/3073/2013. Vaccine effectiveness was analysed for A(H3N2) only, as it was almost the only circulating subtype [30].

In season 2017–2018, a positivity rate of 88.7% was recorded with an estimated 697,000 ILI-visits of which approximately 470,000 were diagnosed with lab-confirmed clinical influenza infection. GPs collected most samples from individuals aged 15–64 years, whereas the least samples were obtained from children <five years and elderly ˃85 years. The highest positivity rates were observed in age categories 5–14 years (69%) and 65–84 years (70%). Distribution of (sub)types varied by age: influenza B/Yamagata dominated in each age group with a ratio of 2:1 and in children 3:1. B/Victoria was detected in individuals <65 years and significantly more in children. A(H1N1)pdm09 was observed more in individuals <65 years (76%), while A(H3N2) infected more elderly ≥ 65 years (60%). SARI collected samples mainly from children <five years old and elderly aged 65–84 years. Children aged 5–14 years (69%) and elderly 65–84 years (70%) had the highest positivity rate. Influenza A subtypes varied within age group, with A(H1N1)pdm09 more prevalent in children and adolescents, while A(H3N2) occurred more in elderly ≥ 65 years. B/Yamagata was present in every age group. Majority of patients with complications were ≥ 65 years, of which 89% had underlying conditions or risk factors. 78% of children and 92% of adults with complications had comorbidities. In children without comorbidities, complications were generally present for <one year. Biggest risk factor for complications was an A(H3N2) infection. Average hospital stay for children <four years was 3.5 days and 13 days for elderly ≥ 85 years. Hospital stay was positively associated with male gender and A(H3N2) infection. Most deaths occurred in elderly ≥ 65 years with risk factors or underlying conditions (75%). Risk of death was associated with being ˃85 years and A(H3N2) infection. Median time between onset of symptoms and death was 14 days. During SARI-surveillance period, GPs had a significant higher positivity rate (66%) compared to hospitals (41.4%). Primary care patients (66%) were significantly more infected with influenza B than hospital patients (60%). The few B/Victoria infections were significantly higher in primary care (3%) than in hospitalisations (1%). Between both surveillances, no significant difference in influenza A subtypes ratio occurred. In both surveillances, some samples could not be (sub)typed due to low viral load [50, 51]. All samples from A(H1N1)pdm09 were represented by vaccine strain A/Michigan/45/2015. Approximately 9% of all circulating viruses in Belgium were A(H3N2), all were represented by vaccine strain A/Hong Kong/4801/2014. It is essential to keep monitoring A(H3N2) as it was evolving quickly. Circulating B/Yamagata viruses belonged to B/Phuket/3073/2013. B/Victoria strains, which primarily belonged to B/Brisbane/60/2008, exhibited antigenic drift and differed from the vaccine virus. All vaccine strains were well-matched with most circulating viruses, except B/Victoria [51]. In term of vaccination status, the general population had a vaccination rate of 22.6%, while the high-risk group achieved 46.2% [52].

Approximately 506,000 Belgians consulted their GP for ILI in season 2018–2019, with 307,000 testing positive for influenza making a positivity rate of 60.7%. Most samples were collected in age group 15–64 years (82%), with very limited samples collected from children <five years and elderly ˃85 years. The positivity rate was comparable across all ages (60–75%), except for children <five years (100%). A(H3N2) was dominant across all age groups, whereas A(H1N1)pdm09 was most common in adults <65 years. Samples collected for SARI-surveillance were primarily from children <five years and elderly between 65–84 years, with positivity rates increasing with age from 13% in children <five years to 39% in those over 65. A(H1N1)pdm09 circulated mainly in children and adolescents, whereas A(H3N2) predominated this season. Off all hospitalised patients, 17% were children, 19% were adults and 61% were elderly ≥ 65 years old. Comorbidities were present in 92% of patients with complications, with chronic respiratory diseases and A(H1N1)pdm09 infection posing the greatest risk. Average hospital stay for children <four years old was 3.6 days and 13.3 days for elderly ≥ 85 years. Hospitalised patients had a higher incidence of dyspnoea (50%) and comorbidities (78%) than patients consulting their GPs (41%-27%). 87.8% of all deaths occurred in individuals aged ≥ 65 years, the other cases were in 15–64 years age group. Overall, ILI-samples (59.8%) were significantly more positive than SARI-samples (33.4%) during SARI-surveillance period. No significant differences in influenza A subtype ratios occurred between both surveillances. Some samples could not be (sub)typed due to low viral load [31, 53]. A quadrivalent vaccine was available during this season, with A(H1N1)pdm09 samples matching the vaccine strain A/Michigan/45/2015. A(H3N2) accounted for 80% of circulating viruses, with most subclades antigenically distinct from reference strain A/Singapore/INFIMH-16–0019/2016. B/Yamagata was not sequenced. B/Victoria virus exhibited antigenic differences from the vaccine virus, resulting in non-significant vaccine protection (1%). The vaccine was 75% effective against A(H1N1)pdm09, with significant protection against hospitalisations (80%) but not against infections requiring a GP consultation (50%) [53]. Only one strain showed reduced sensitivity to.

## Discussion

When interpreting results, it is crucial to take into account the difference in surveillance period between ILI- and SARI-surveillance, as this can influence the observed positivity rates due to variations in case definitions. ILI-surveillances include all patients with flu-like symptoms based on specific criteria, including sudden onset of symptoms, high fever and respiratory and systemic symptoms [29–31]. This ensures the broader inclusion of individuals in the surveillance program over an extended duration. ILI-surveillances, except for the 2009 pandemic when the follow up was around 13 weeks longer, collected all respiratory samples within a similar timeframe. Since 2011, the start of the surveillance period has remained consistent. However, in the last three seasons, the surveillance period ended earlier than usual (see Table 3). In contrast, SARI-surveillances relied on an estimation of ILI-data and hence, had a more fluctuating period of data collection. The criteria utilised to determine a SARI encompass a manifestation onset within seven days, fever of ≥ 38 °C, cough or dyspnea that necessitated hospitalisation for at least 24 h. This surveillance primarily targeted severe influenza cases, and therefore executed exclusively during the seasonal influenza epidemic period [29–31]. The consequence of this approach was the inclusion of a more restricted group of individuals for a relatively shorter data collection duration. Typically, it commences when influenza is detected in ILI-samples and incidence of ILI starts to rise, and ends at least three weeks after the conclusion of the influenza epidemic. However, it is worth noting that season 2011–2012 (05/2012–16/2012) and season 2014–2015 (06/2015–16/2015) reporter data for a shorter period than the surveillance was active. During 2018–2019 season, a pilot study was conducted in which three of six hospitals initiated surveillance earlier to analyse the respiratory syncytial virus. In week 2/2019 all six hospitals commenced data collection.

**Table 3:** Time period of both surveillances

Year	ILI (Week/year)	SARI (Week/year)
’09–‘10	30/2009–23/2010	/
’10–‘11	35/2010–20/2011	40/2010–20/2011
’11–‘12	40/2011–20/2012	03/2012–16/2012
’12–‘13	40/2012–20/2013	51/2012–19/2013
’13–‘14	40/2013–20/2014	06/2014–16/2014
’14–‘15	40/2014–20/2015	52/2014–17/2015
’15–‘16	40/2015–20/2016	01/2016–17/2016
’16–‘17	40/2016–12/2017	01/2017–17/2017
’17–‘18	40/2017–18/2018	50/2017–18/2018
’18–‘19	40/2018–18/2019	40/2018–14/2019
Median	40–20	6–17
Modus	40–20	/–16

Median and modus are calculated with all available data

Over the years, vaccines have typically included two A subtypes and one B lineage, with a 50% probability of selecting the dominant lineage given that both B-viruses were in circulation most seasons [54, 55]. A quadrivalent vaccine that includes both A- and B-viruses has been developed to provide a broader protection. In 2013, the WHO published its first set of guidelines recommending the inclusion of both expected B-strains in the vaccine [56]. Since 2015–2016 season, this quadrivalent vaccine has been available in Belgium [47].

WHO issues recommendations for influenza vaccine compositions based on circulating viruses, several months in advance [11, 54, 56]. It is crucial for vaccine strains to match antigenically with circulating viruses [11, 54]. A mismatch may result in a less effective vaccine and potential epidemic outbreak as it is not possible to produce and distribute a new vaccine in time [4, 11, 54]. The pandemic of 2009 highlighted this issue, as the seasonal vaccine did not protect against dominant A(H1N1)pdm09 subtype, and the Pandemric vaccine proved effective against it [20]. A mutation (as seen in the H3N2-virus strain in season 2013–2014 or the egg propagation of vaccine seed virus in season 2018–2019) or an antigenic drift (like in H3 in season 2014–2015) can negatively impact vaccine effectiveness. Over the past decade, the vaccine has been less than 50% effective in most seasons, highlighting the need for more comprehensive, durable and rapidly producible vaccines.

## Conclusion

The perceived triviality of influenza often underestimates its significant impact, emphasizing the need for vigilant monitoring of the virus on a seasonal basis. Influenza A was found to be the predominant strain in both primary care and hospital settings, with 73.7% [± 27.5] of positive samples in the ILI-surveillance and 77.7% [± 23.8] in the SARI-surveillance. During this ten-year period, A(H3N2) within influenza A and B/Yamagata-lineage within influenza B were predominant. The vaccine effectiveness varied notable, with an average of 34.9% [± 15.3] between 2012 and 2019.

Both surveillance systems collect information to detect early warning signs of an impending epidemic or pandemic, estimate severity of illness for healthcare planning purposes and make informed decisions about vaccination composition. Despite these efforts, the evolving nature of the influenza virus makes it a challenging threat to identify and contain. Influenza prevention, control and treatment must be improved to reduce the given threat. A potential solution to combat influenza more effectively is to develop vaccines that provide broader and more durable protection that can also be produced more rapidly. Including more subtypes and lineages in vaccine compositions could enhance vaccine effectiveness by reducing the likelihood of mismatches, which in turn could mitigate the severity of epidemics and pandemics.

## Data Availability

Not applicable.
